# Comparison of faecal protein biomarkers' diagnostic accuracy for colorectal advanced neoplasms: a systematic review and meta-analysis

**DOI:** 10.1038/s41598-022-06689-4

**Published:** 2022-02-16

**Authors:** Atefeh Nasir Kansestani, Mohammad Erfan Zare, Qingchao Tong, Jun Zhang

**Affiliations:** Department of Clinical Laboratory, School of Medicine, Sir Run Run Shaw Hospital, Zhejiang University, Hangzhou, China

**Keywords:** Biochemistry, Cancer, Gastroenterology, Oncology

## Abstract

Early diagnosis of colorectal advanced neoplasms (ANs), including colorectal cancer (CRC) and advanced adenoma (AA), has a positive effect on the survival rate. As a first attempt, the aim of this meta-analysis was to compare the diagnostic accuracy of faecal protein biomarkers for the detection of colorectal neoplasms with consideration of a wide range of covariates. A systematic literature search was performed up to Jun 10, 2021 on Web of Sciences, Scopus and PubMed. The diagnostic accuracies were calculated using the bivariate/hierarchical random effect model. Biomarkers were determined to be clinically applicable (CA) if they had areas under the curve > 0.70 and positive and negative likelihood ratios > 2 and < 0.5, respectively. A total of 47,059 test results were extracted from 16 immunochemical faecal occult blood test (iFOBT), 26 pyruvate kinase-M2 (PK-M2) and 23 faecal calprotectin (FC) studies. Only iFOBT, PK-M2 and FC for CRC plus iFOBT and PK-M2 for AN were CA. iFOBT had significantly superior accuracy (P = 0.02 versus PK-M2 and P < 0.01 versus FC for CRC; P < 0.01 versus PK-M2 for AN). Regarding covariates, the lateral flow method of PK-M2 measurement increased its accuracy for CRC detection compared to the enzyme-linked immunosorbent assay (P < 0.01). iFOBT is recommended as the most accurate faecal biomarker for CRC and AN diagnosis.

## Introduction

Colorectal cancer (CRC) is currently the third-most prevalent malignancy and the second leading cause of death among cancerous patients^1^. Despite the fulfilment of major efforts such as screening programs, the increasing trend of new cases in recent years indicates that better strategies are required not only for the early diagnosis of CRC but also for other types of colorectal advanced neoplasms (ANs) as important precursors of CRC. AN includes CRC and advanced adenoma (AA). AA is defined as multiple colorectal polyps or individual lumps ≥ 1 cm in size, tubulovillous or villous histology features or high-grade dysplasia. AA patients have a higher risk of developing CRC^2^.

Early diagnosis of AN has a positive correlation with a high survival rate owing to implementation of proper treatments, especially in high-risk groups, including first-degree relatives (FDRs) of individuals with CRC and AA. Guidelines from different authoritative societies recommend AN screening for average-risk individuals by age 50 years or older and 40 or 10 years for high-risk groups. Today, colonoscopy is considered the gold standard for AN diagnosis and screening^2,3^. However, colonoscopy is an expensive, invasive and operator skill-dependent technique. In addition, it requires unpleasant bowel preparation and occasionally causes serious complications. Therefore, implementing noninvasive biomarkers for the diagnosis of AN seems to be necessary^4^.

Today, a wide range of faecal biomarkers consisting of stool DNA testing, stool miRNAs, the faecal microbiome and different proteins have been introduced for the diagnosis and screening of AN. However, faecal protein biomarkers have special importance due to their low cost, noninvasiveness and simple sampling procedure attributes^5^. The first introduced faecal biomarker for AN was the guaiac-based faecal occult blood test (gFOBT), and since its introduction, it has saved many human lives, despite its low sensitivity. This method has been replaced by the immunochemical faecal occult blood test (iFOBT), which has much higher sensitivity^2,5^. In recent decades, some novel and promising faecal protein biomarkers have been introduced for the diagnosis and screening of CRC and other ANs. For example, pyruvate kinase-M2 (PK-M2) with an overall accuracy of 0.85^6^ and faecal calprotectin (FC) with an overall accuracy of 0.81^7^ have been reported for the detection of CRC in the latest published systematic review and meta-analyses. Nonetheless, there is no comparative systematic review or meta-analysis to find the most accurate faecal protein biomarker. Given the above information, as a first attempt, the aim of this evidence-based meta-analysis was to compare the diagnostic accuracy of clinically available faecal protein biomarkers for the detection of CRC, AA and AN with consideration of a wide range of covariates to find and recommend the most accurate one.

## Methods

### Search strategy

The search strategy of the present systematic review was carried out based on the Preferred Reporting Items for Systematic Reviews and Meta-Analyses (PRISMA) statement^8^. We performed systematic searches on electronic databases containing Web of Science, Scopus and MEDLINE/PubMed until June 10, 2021, without any language restrictions. Additionally, the Chinese National Knowledge Infrastructure (CNKI) database for Chinese full text articles and the Scientific Information Database (SID) database for Persian full text articles were searched. The following MeSH terms (“Colorectal neoplasms”) AND (“Diagnosis” OR “Early detection of cancer”) were used to search MEDLINE/PubMed, and text words containing (“Colorectal cancer” OR “CRC” OR “Colorectal malignancy” OR “Colorectal tumour” OR “Adenoma” OR “Colorectal neoplasms”) AND (“Faecal biomarker” OR “Laboratory tests” “Diagnostic biomarker” OR “Screening Biomarker”) were used to search other databases, besides MEDLINE/PubMed (Supplemental Table S1). Finally, similar papers which were purposed by Pubmed, as well as google scholar, the reference lists of each selected paper and related systematic and narrative reviews on this topic were assessed to identify missed studies. To exclude duplicate papers, records were imported into EndNote software (Version X9, Thomson Reuters).

### Study selection and data extraction

Two reviewers (A.N. K and M.E. Z) independently screened the title and abstract of all obtained records for eligibility and inclusion. The inclusion criteria were as follows: (1) patients for whom a faecal protein biomarker was used to detect CRC, AA or AN; (2) CRC and AA should be confirmed by colonoscopy and pathology reference standards; (3) specific diagnostic information was sufficient to construct a 2 × 2 contingency table; and (4) for each faecal biomarker, at least 4 studies should be found. Exclusion criteria were set as follows: (1) duplicated studies, review articles, editorials, case reports, and clinical guidelines; (2) insufficient data reporting to construct the 2 × 2 contingency table; CRC and colorectal AA were not verified by the aforementioned reference standards.

A custom-made form was utilized for data extraction, including the first author’s name, publication year, country of the study, subjects’ average age, gender, study design, total sample size, true positives, true negatives, false-positives, and false negatives. The results of iFOBT were extracted in those studies, which was accomplished along with other assessed biomarkers. To achieve more reliable results in case–control designed studies, 2 × 2 contingency tables were constructed by comparing the specific characteristics versus not only healthy controls but also other patients, which did not have those specific characteristics. To homogenize different units, mg/L (= μg/mL) was transformed to μg/g by multiplying each value by a factor of 5.

In CRC patients, the percentages of distal and late-stage tumours were extracted. Proximal tumours were defined as those located from the caecum to the transverse colon, and distal tumours were located from the splenic flexure to the rectum. In addition, late-stage tumours were defined as CRC stages III + IV or Dukes’ stages C + D versus 0 + I + II or Dukes’ stages A + B, which were categorized as early-stage tumours^9^. Colorectal adenomas were defined as AA when the following features were present: (1) high-grade dysplasia; (2) tubulovillous or villous components; and (3) multiple adenomas or individual lumps ≥ 1 cm in size. AN included CRC and/or AA.

### Quality assessment and publication bias

The methodological quality of each included study was assessed utilizing the Quality Assessment of Diagnostic Accuracy Studies-2 (QUADAS-2) tool. QUADAS-2 evaluates four key domains made up of “patient selection”, “index test”, “reference standard”, and “flow and timing” in two categories, “risk of bias” for all four domains and “applicability” for the first three domains in the diagnostic accuracy studies. Each category was scored as low, high or unclear according to the assessment criteria. All disagreements were resolved by consensus after discussion. Furthermore, to evaluate potential publication bias, the linear regression method was utilized to assess the asymmetry of Deeks’ funnel plot. P < 0.1 for the slope coefficient reveals the presence of publication bias.

### Statistical analysis

To construct a 2 × 2 contingency table, true positives, false positives, true negatives and false negatives were calculated for each included study. A standard bivariate method was employed to calculate the summary points, including pooled sensitivity, pooled specificity, pooled positive likelihood ratio (PLR +), pooled negative likelihood ratio (PLR−) and pooled diagnostic odds ratio (PDOR). Using a hierarchical model, summary receiver operating characteristic (HSROC) curves were plotted to determine the area under the curve (AUC) as a global measure of test performance. The overall diagnostic accuracy of each biomarker was interpreted according to AUC, PLR+ and PLR−. The relationship between the AUC value and diagnostic accuracy is described as follows: 0.5–0.70 is interpreted as not acceptable, 0.71 to 0.79 acceptable, 0.80–0.89 good and 0.90–1 excellent. Additionally, based on PLR+ and PLR−, the diagnostic accuracy of each biomarker is divided into four categories. PLR− values < 0.1, 0.1–0.2, 0.2–0.5 and > 0.5 represent substantial, moderate, small and nonmeaningful evidence, respectively, to rule out disease existence. PLR+ values > 10, 5–10, 2–5 and < 2 are considered substantial, moderate, small and not meaningful evidence to rule in disease existence, respectively. The results of LRs were summarized by a scattergram.

In this study, we considered clinically applicable biomarkers if they had AUC > 0.70, PLR+ > 2, and PLR− < 0.5. To compare the diagnostic accuracy of different clinically applicable biomarkers, relative DORs (RDORs) and their P values were computed.

Between-study heterogeneity was evaluated using Higgins’ inconsistency index (I^2^). I^2^ > 50% implied substantial heterogeneity. To find potential sources of heterogeneity and explore the robustness of the results, when sufficient studies were available, subgroup analysis was performed based on the method of measurement, cut off, study type and QUADAS-2 domains. Additionally, meta-regression analysis was carried out on age and sex covariates for all neoplasms as well as tumour location and stage covariates for CRC. In addition, to illustrate another potential source of heterogeneity, the Spearman correlation coefficient was calculated to determine the threshold effect.

In the present study, calculations were conducted and summarized for reporting considering a 95% confidence interval (95% CI), and reports were defined as statistically significant when P < 0.05 (except publication bias). All statistical analyses were performed by “midas” commands in Stata software (Stata Corporation, College Station, TX, USA, version 12.0), and RevMan 5.3 was employed to draw comparative HSROC plots.

## Results

### Study selection

**Figure 1:**
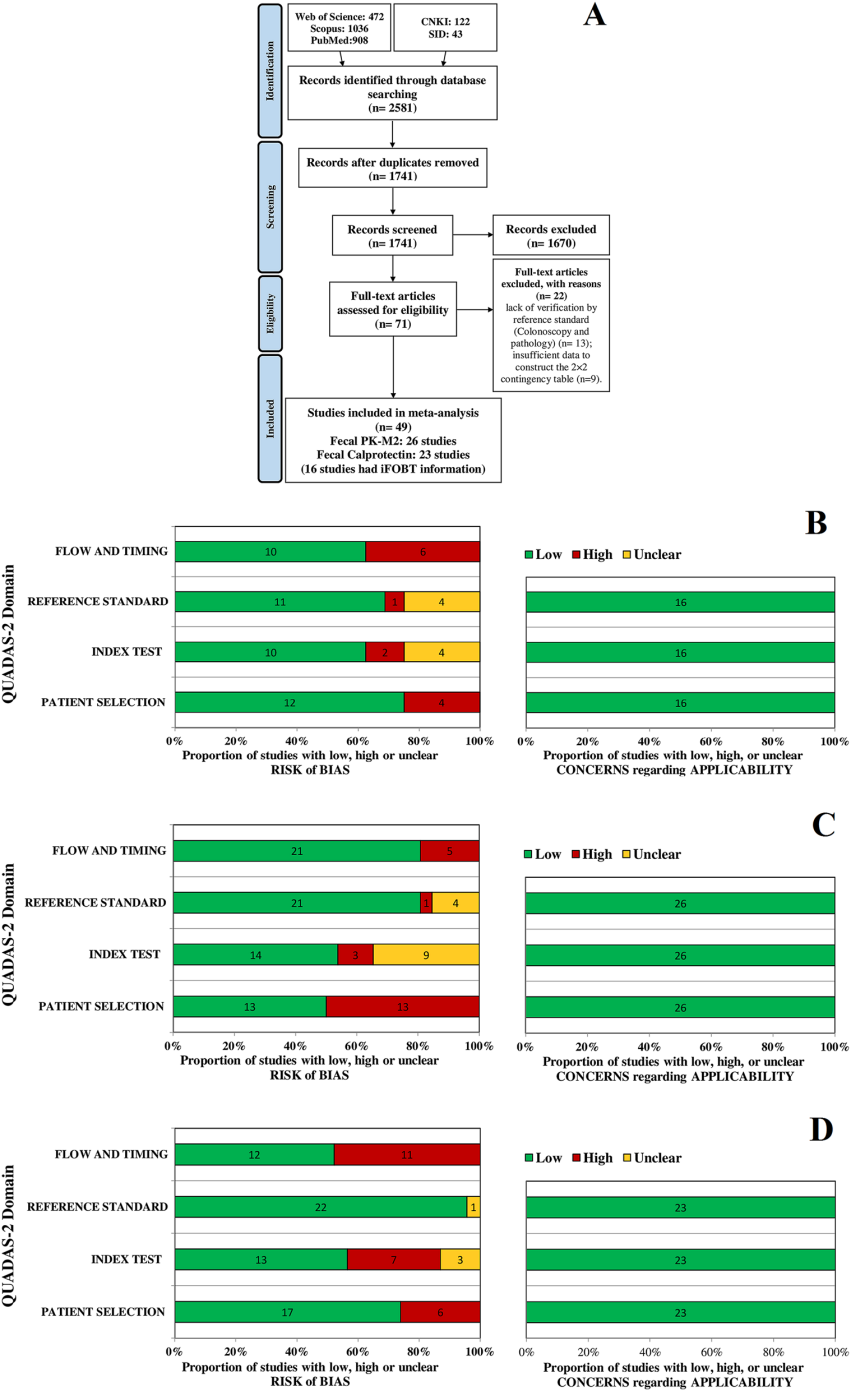
Flowchart diagram of study selection and quality assessment of included studies utilizing the QUADAS-2. (**A**) Flowchart diagram of study selection based on the inclusion and exclusion criteria; (**B**) QUADAS-2 diagram for iFOBT; (**C**) QUADAS-2 diagram for PK-M2; (**D**) QUADAS-2 diagram for FC. *iFOB* immunochemical faecal occult blood tests, *PK-M2* pyruvate kinase-M2, *FC* faecal calprotectin, *QUADAS-2* quality assessment of diagnostic accuracy studies-2.

Among 2581 initial records, 840 studies were excluded owing to duplication, and 1670 were excluded after screening the title and abstract. In this stage, the most common reasons for exclusion were (1) review articles, editorials, case reports, and clinical guidelines; (2) laboratory biomarkers evaluated on nonfaecal samples (serum and tissue); and (3) nonprotein biomarkers such as molecular biomarkers and microbiome mass. Finally, 71 studies underwent full text assessment. Among these, 22 studies were excluded due to the following reasons: (1) lack of verification by reference standard (colonoscopy and pathology) (n = 13) and (2) insufficient data to construct the 2 × 2 contingency Table (n = 9). Eventually, 49 studies with 47,059 test results were included in the present study (Fig. 1A).

Pursuant to the inclusion criteria, PK-M2 and FC were found to be eligible biomarkers for further assessment.

Sixteen of 49 included studies reported iFOBT data in addition to the other assessed biomarkers, with 13,769 test results^10–25^. All 16 studies had iFOBT results for the diagnosis of CRC (5610 test results), 10 studies had results for AA (4008 test results) and 11 studies had results for AN (4151 test results). One study evaluated iFOBT with two different commercial kits, so we constructed two separated 2 × 2 contingency tables from this article^16^.

From 26 PK-M2 included studies with 12,213 test results^13–22,26–41^, 25 studies reported the results of CRC detection (5706 test results), 10 studies for AA (3781 test results) and 10 studies for AN (2726 test results). One study assessed PK-M2 by two different methods and therefore built two 2 × 2 contingency tables from this article^13^.

We found 23 studies with 21,077 test results for FC^10–12,19,20,23–25,30,42–55^, all of which had information on CRC diagnosis with 9747 test results. The FC results for the detection of AA and AN were extracted from 9 articles with 5665 test results for each condition. There were two studies that evaluated FC by two different methods, so two separate 2 × 2 contingency tables were constructed for each article^25,44^.

Two studies evaluated all three biomarkers^19,20^, and one study evaluated PK-M2 and FC at the same time for CRC diagnosis^30^. Table 1 summarizes the main characteristics of the included studies in this review.

**Table 1:** Characteristics of included studies.

Study ID	Language	Country	Study design^e^	Study population			Number of populations			Cut-off Value	Method^d^	%Distal	%Late
				Sample	%Male	Age	CRC^a^	AA^b^	AN^c^				
**Immunochemical faecal occult blood tests (iFOBT)**													
Turvill, 2018^10^	English	UK	C	515	50	69	27	–	–	7 µg/g	LAIT	NA^f^	NA
Högberg, 2017^11^	English	Sweden	C	373	36.4	63	8	8	16	25 µg/g	Lateral flow	NA	NA
Zaccaro, 2017^18^	English	Italy	C	127	50.3	63	–	–	11	20^i^ µg/g	LAIT	NA	NA
Widlak, 2017^23^	English	UK	C	430	49	67	24	1	5	7 μg/g	LAIT	76	NA
Caviglia, 2016^21^	English	Italy	C	572	66.2	66	7	19	26	20 μg/g	LAIT	NA	NA
Cho, 2016^22^	English	Korea	C	236	67.8	47	1	3	4	7^i^ μg/g	LAIT	NA	NA
Rutka, 2016^19^	English	Hungary	C	95	40	67	19	–	–	3^i^ μg/g	Lateral flow	89	31.5
Kim, 2015^13^	English	Korea	CC	323	57	62	139	–	–	10^i^ μg/g	Lateral flow	NA	55.4
Mowat, 2016^24^	English	UK	C	755	45.3	64	28	40	68	10 μg/g	LAIT	NA	NA
Kok, 2012^25^	English	Netherland	C	382	46.3	60	19	16	35	6 μg/g	Lateral flow	NA	NA
Parente, 2012^20^	English	Italy	C	280	56	67	47	85	132	20^i^ μg/g	LAIT	NA	NA
Karl, 2008^12^	English	Germany	CC	551	47.2	65.6	186	–	–	12.27 μg/g	ELISA	NA	NA
Shastri, 2008^15^	English	Germany	C	640	41.4	52.6	55	21	76	2^i^ μg/g	Lateral flow	61.8	34.5
Mulder^h^, 2007^16^	English	Netherland	C	181	50.2	58	52	22	74	30 μg/g	Lateral flow	65	81^ g^
Mulder^h^, 2007^16^	English	Netherland	C	181	50.2	58	52	22	74	10 μg/g	Lateral flow	65	81^ g^
Guan-Fu, 2006^14^	Chinese	China	CC	86	NA	NA	43	–	–	2^i^ μg/g	Lateral flow	74.4	51.1
Vogel, 2005^17^	German	Germany	CC	138	44.2	58	22	–	–	30 μg/g	Lateral flow	NA	NA
**Pyruvate kinase-M2 (PK-M2)**													
Alhadi, 2021^26^	English	Malaysia	C	85	58.8	56.8	17	–	–	4 U/mL	Lateral flow	NA	NA
Rigi, 2020^27^	English	Iran	C	226	NA	NA	39	–	–	4 U/mL	ELISA	53.8	NA
Dabbous, 2019^28^	English	Egypt	CC	60	71.6	52	20	–	–	4 U/mL	ELISA	NA	NA
Zaccaro, 2017^18^	English	Italy	C	127	50.3	63	–	–	11	4 U/mL	ELISA	NA	NA
Caviglia, 2016^21^	English	Italy	C	572	66.2	66	7	19	26	4 U/mL	ELISA	NA	NA
Cho, 2016^22^	English	Korea	C	236	67.8	47	1	3	4	4 U/mL	Lateral flow	NA	NA
Rutka, 2016^19^	English	Hungary	C	95	40	67	19	20	39	4 U/mL	Lateral flow	89	31.5
Kim, 2015^13^	English	Korea	CC	323	57	62	40	–	–	4 U/mL	ELISA	NA	NA
Kim, 2015^13^	English	Korea	CC	323	57	62	139	94	233	4 U/mL	Lateral flow	NA	55.4
Sithambaram, 2015^29^	English	Malaysia	CC	300	52.7	62.5	100	–	–	4 U/mL	Lateral flow	86	NA
Wang, 2014^30^	English	China	CC	40	60	67.5	19	–	–	114 U/ml	ELISA	65	55
Wei, 2014^31^	Chinese	China	CC	134	61.2	55.2	74	–	–	166.7 µkat/L	ELISA	NA	NA
Abdullah, 2012^32^	English	Indonesia	C	328	60.1	53.4	42	–	–	4 U/mL	ELISA	NA	NA
Parente, 2012^20^	English	Italy	C	280	56	67	47	85	132	4 U/mL	ELISA	NA	NA
Li, 2011^33^	Chinese	China	CC	66	NA	NA	44	–	–	4 U/mL	ELISA	NA	NA
Haug, 2008^34^	English	Germany	C	1082	50	63	–	106	–	4 U/mL	ELISA	NA	NA
Shastri, 2008^15^	English	Germany	C	640	41.4	52.6	55	21	76	4 U/mL	ELISA	61.8	34.5
Koss, 2008^36^	English	UK	CC	55	67.2	66.3	32	5	37	4 U/mL	ELISA	NA	37.5
Haug, 2007^35^	English	Germany	CC	982	44.2	63.5	65	–	–	4 U/mL	ELISA	75.3	58.4
Zhang, 2007^37^	Chinese	China	CC	95	73.6	48.6	31	–	–	4 U/mL	ELISA	NA	54.8
Mulder, 2007^16^	English	Netherland	C	181	50.2	58	52	22	74	4 U/mL	ELISA	65	81^ g^
Guan-Fu, 2006^14^	Chinese	China	CC	86	NA	NA	43	–	–	4 U/mL	ELISA	74.4	51.1
Shastri, 2006^38^	English	Germany	C	317	47.9	56	74	10	84	4 U/mL	ELISA	NA	NA
Tonus, 2006^39^	English	Germany	CC	96	56.2	66	54	–	–	4 U/mL	ELISA	NA	NA
Vogel, 2005^17^	German	Germany	CC	138	44.2	58	22	–	–	4 U/mL	ELISA	NA	NA
Naumann, 2004^40^	German	Germany	C	232	NA	NA	27	–	–	4 U/mL	ELISA	NA	NA
Hardt, 2004^41^	English	Germany	CC	204	NA	NA	60	–	–	4 U/mL	ELISA	NA	55
**Faecal calprotectin**													
Turvill, 2018^10^	English	UK	C	515	50	69	27	–	–	118 µg/g	ELISA	NA	NA
Högberg, 2017^11^	English	Sweden	C	373	36.4	63	8	8	16	50 µg/g	ELISA	NA	NA
Rutka, 2016^19^	English	Hungary	C	95	40	67	19	–	–	128.5 μg/g	ELISA	89	31.5
Turvill, 2016^55^	English	UK	C	654	44	69	39	–	–	50 μg/g	ELISA	NA	NA
Widlak, 2017^23^	English	UK	C	430	49	67	24	1	25	50 µg/g	ELISA	76	NA
Mowat, 2016^24^	English	UK	C	755	45.3	64	28	41	69	50 μg/g	ELISA	NA	NA
Khoshbaten, 2014^42^	English	Iran	CC	100	65	47	50	–	–	75.8 µg/g	ELISA	NA	NA
Wang, 2014^30^	English	China	CC	40	60	67.5	19	–	–	144 IU/ml	ELISA	65	55
Kok, 2012^25^	English	Netherland	C	382	46.3	60	19	16	35	50 µg/g	ELISA	NA	NA
Kok, 2012^25^	English	Netherland	C	382	46.3	60	19	16	35	50 µg/g	Lateral flow	NA	NA
Parente, 2012^20^	English	Italy	C	280	56	67	47	85	132	50 μg/g	ELISA	NA	NA
Meucci, 2010^43^	English	Italy	C	870	47.5	59.1	21	–	–	50 µg/g	ELISA	NA	NA
Damms, 2008^44^	English	Germany	CC	140	44.2	58	8	–	–	50 µg/g	ELISA	NA	NA
Damms, 2008^44^	English	Germany	CC	140	44.2	58	8	–	–	50 µg/g	Lateral flow	NA	NA
Karl, 2008^12^	English	Germany	CC	551	47.2	65.6	186	–	–	50 µg/g	ELISA	NA	NA
Hoff, 2004^45^	English	Norway	C	2321	49	58	16	195	206	50 µg/g	ELISA	NA	NA
Carroccio, 2003^46^	English	Italy	C	80	43.7	62.5	3	–	–	50 μg/g	ELISA	NA	NA
Costa, 2003^47^	English	Italy	CC	239	46.4	46.3	18	8	26	50 μg/g	ELISA	NA	NA
Summerton, 2002^48^	English	UK	C	134	NA	NA	8	–	–	50 μg/g	ELISA	NA	NA
Tibble, 2002^49^	English	UK	C	346	NA	NA	7	–	–	50 μg/g^i^	ELISA	NA	NA
John, 2001^50^	English	Norway	C	453	51	66	154	–	–	50 μg/g	ELISA	NA	NA
Kristinsson, 2001^51^	English	Norway	C	253	39.5	60	5	4	9	50 μg/g^i^	ELISA	NA	NA
Tibble, 2001^52^	English	UK	C	295	NA	NA	66	22	88	50 μg/g^i^	ELISA	NA	NA
Tibble, 2000^53^	English	UK	C	220	28.6	43	2	–	–	50 μg/g^i^	ELISA	NA	NA
Røseth, 1993^54^	English	Norway	CC	206	NA	61.6	53	–	–	50 μg/g^i^	ELISA	66	NA

^a^Colorectal cancer.

^b^Advanced adenoma.

^c^Advanced neoplasms.

^d^*LAIT* latex agglutination immunoturbidimetry, *ELISA* enzyme-linked immunosorbent assay.

e*C* cohort, *CC* case–control.

^f^Not available.

^g^Five patients did not undergo colorectal resection. In those cases, tumour invasion could not be determined.

^h^iFOB was performed by two different commercial kits.

^i^This unit was transformed to μg/g.

### Quality assessment and publication bias

The quality of the included studies was assessed using the QUADAS-2 tool, and the results were illustratively summarized for each biomarker (Fig. 1B–D). The quality assessment results of the included studies in the iFOBT group revealed the major risk of bias in the “flow and timing” and “patient selection” categories mainly because all patients were not included in the analysis and case–control study design, respectively (Fig. 1B). Regarding PK-M2 included studies, the major risk of bias occurred in the “patient selection” category because of the case–control study design. Additionally, there were 3 studies with high risk and 9 studies with unclear risk of bias in the “index test” category as a result of a lack of prespecified thresholds and unclear index test interpretation without knowledge of the reference standard result (Fig. 1C). Concerning FC included studies, the greatest risk of bias referred to “flow and timing” and “index test” owing to the aforementioned reasons (Fig. 1D). The included studies for all biomarkers raised no concerns regarding applicability.

Table 2 includes the publication bias analyses of each group. Regarding CRC diagnosis, Deeks’ funnel plot asymmetry test indicated that there was no significant publication bias in the iFOBT, PK-M2 and FC biomarker datasets (Supplemental Fig. S1A–C). In relation to AA detection, significant publication bias in the iFOBT dataset and the absence of publication bias in the PK-M2 and FC datasets were found (Supplemental Fig. S2A–C). Concerning AN diagnosis, analyses indicated no significant publication bias in iFOBT and PK-M2 but indicated significant publication bias in FC datasets (Supplemental Fig. S3A–C).

**Table 2:** Diagnostic accuracy of faecal biomarkers and their comparisons.

Test^b^	P-Se (95% CI) (% I^2^)	P-Sp (95% CI) (% I^2^)	P-LR+ (95% CI)	P-LR− (95% CI)	P-DOR (95% CI)	AUC (95% CI)	TE	PB	CA^a^
**Diagnostic accuracy of faecal protein biomarkers**									
**Colorectal cancer**									
iFOBT	0.83 (0.74–0.89) (87.6%)	0.86 (0.81–0.90) (95.2%)	6.10 (4.5–8.2)	0.20 (0.13–0.31)	30 (18–49)	0.91 (0.89–0.94)	0.32	0.72	A
PK-M2	0.82 (0.76–0.87) (82.1%)	0.73 (0.65–0.80) (95.1%)	3 (2.3–4.0)	0.24 (0.18–0.33)	12 (8–20)	0.85 (0.82–0.88)	0.51	0.73	A
FC	0.85 (0.80–0.89) (77.9%)	0.65 (0.57–0.71) (97.5%)	2.4 (2.0–2.9)	0.23 (0.18–0.30)	10 (7–15)	0.85 (0.81–0.87)	0.40	0.53	A
**Advanced adenoma**									
iFOBT	0.53 (0.35–0.70) (82.3%)	0.81 (0.70–0.89) (97%)	2.8 (1.7–4.4)	0.58 (0.41–0.83)	5 (2–10)	0.74 (0.70–0.78)	0.66	0.05	NA
PK-M2	0.46 (0.34–0.58) (84.7%)	0.64 (0.48–0.77) (98.4)	1.3 (0.8–2.0)	0.85 (0.64–1.13)	1 (1–3)	0.54 (0.49–0.58)	0.33	0.83	NA
FC	0.45 (0.35–0.55) (84.2%)	0.56 (0.45–0.66) (98.5%)	1.0 (0.9–1.2)	0.99 (0.89–1.10)	1 (1–1)	0.50 (0.45–0.54)	0.58	0.10	NA
**Advanced neoplasm**									
iFOBT	0.72 (0.58–0.83) (90.2%)	0.88 (0.80–0.92) (96%)	5.9 (4.1–8.4)	0.31 (0.21–0.47)	19 (12–28)	0.88 (0.85–0.91)	0.49	0.53	A
PK-M2	0.68 (0.63–0.73) (56.72%)	0.76 (0.65–0.84) (95.9%)	2.9 (2.0–4.2)	0.42 (0.36–0.48)	7 (4–11)	0.73 (0.69–0.77)	< 0.01	0.22	A
FC	0.70 (0.58–0.80) (92.3%)	0.59 (0.49–0.70) (98.3%)	1.7 (1.4–2.2)	0.50 (0.36–0.70)	3 (2–6)	0.69 (0.65–0.73)	0.78	0.01	NA

Comparison	RDOR (95% CI)	P value							
**Comparison of faecal biomarker diagnostic accuracies**									
**Colorectal cancer**									
iFOBT versus PK-M2	2.49 (1.12–5.54)	0.02							
iFOBT versus FC	2.96 (1.50–5.85)	< 0.01							
PK-M2 versus FC	1.07 (0.56–2.05)	0.83							
**Advanced neoplasm**									
iFOBT versus PK-M2	2.84 (0.88–9.21)	0.07							
iFOBT versus FC	3.44 (1.22–9.68)	0.02							
PK-M2 versus FC	1.03 (0.35–3.04)	0.95							
**Advanced neoplasm**									
iFOBT versus PK-M2	2.70 (1.42–5.12)	< 0.01							
iFOBT versus FC	6.41 (2.75–14.96)	< 0.01							
PK-M2 versus FC	1.70 (0.75–3.83)	0.19							

^a^*P-Se* pooled-sensitivity, *P-Sp* pooled-specificity, *P-LR* pooled-likelihood ratio, *P-DOR* pooled-diagnostic odds ratio, *AUC* area under the curve, *TE* P value of threshold effect, *PB* P value of publication bias, *CA* clinical applicability.

^b^*iFOB* immunochemical faecal occult blood tests, *PK-M2* pyruvate kinase-M2, *FC* faecal calprotectin, *A* applicable, *NA* not applicable, *RDOR* relative DOR.

### Diagnostic accuracy of faecal biomarkers

**Figure 2:**
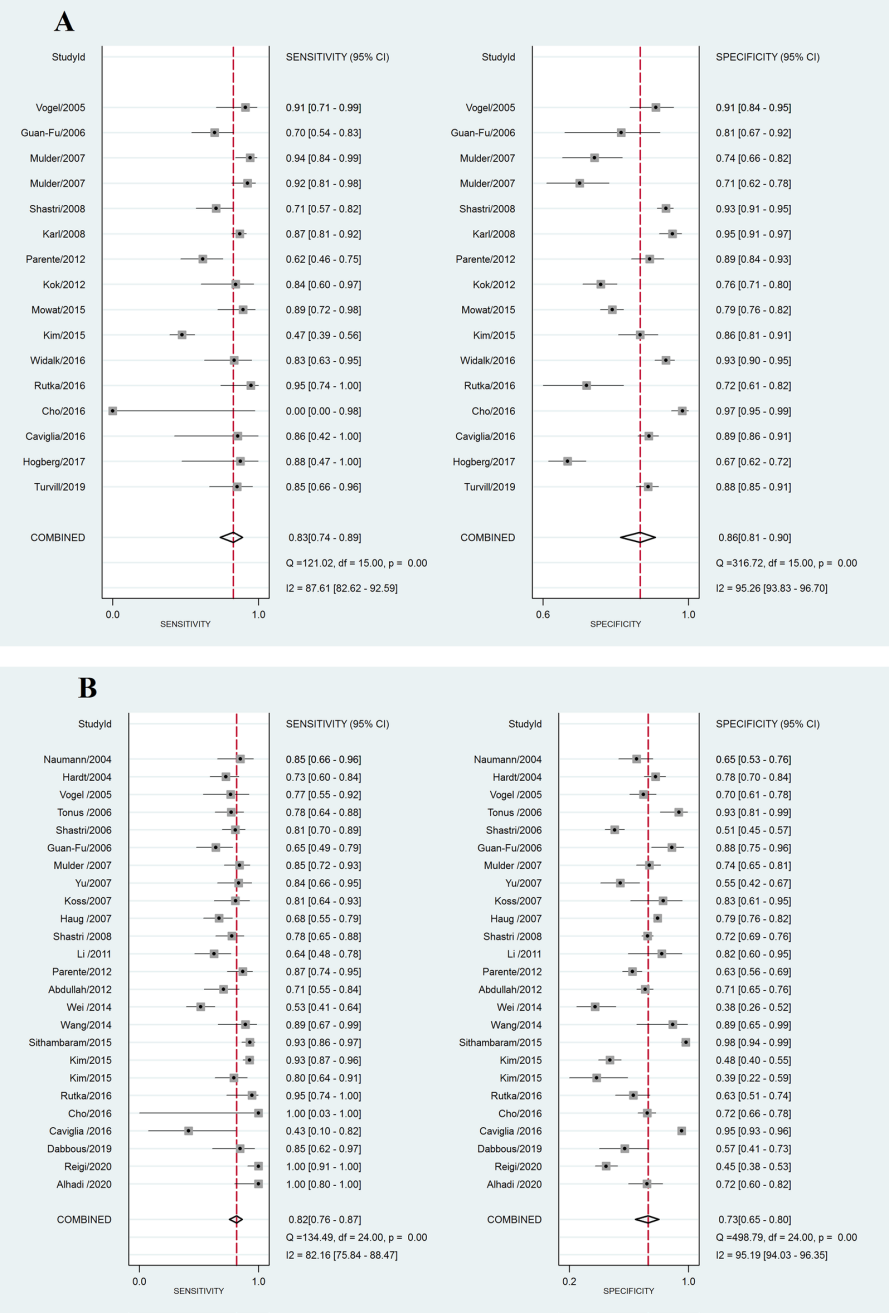

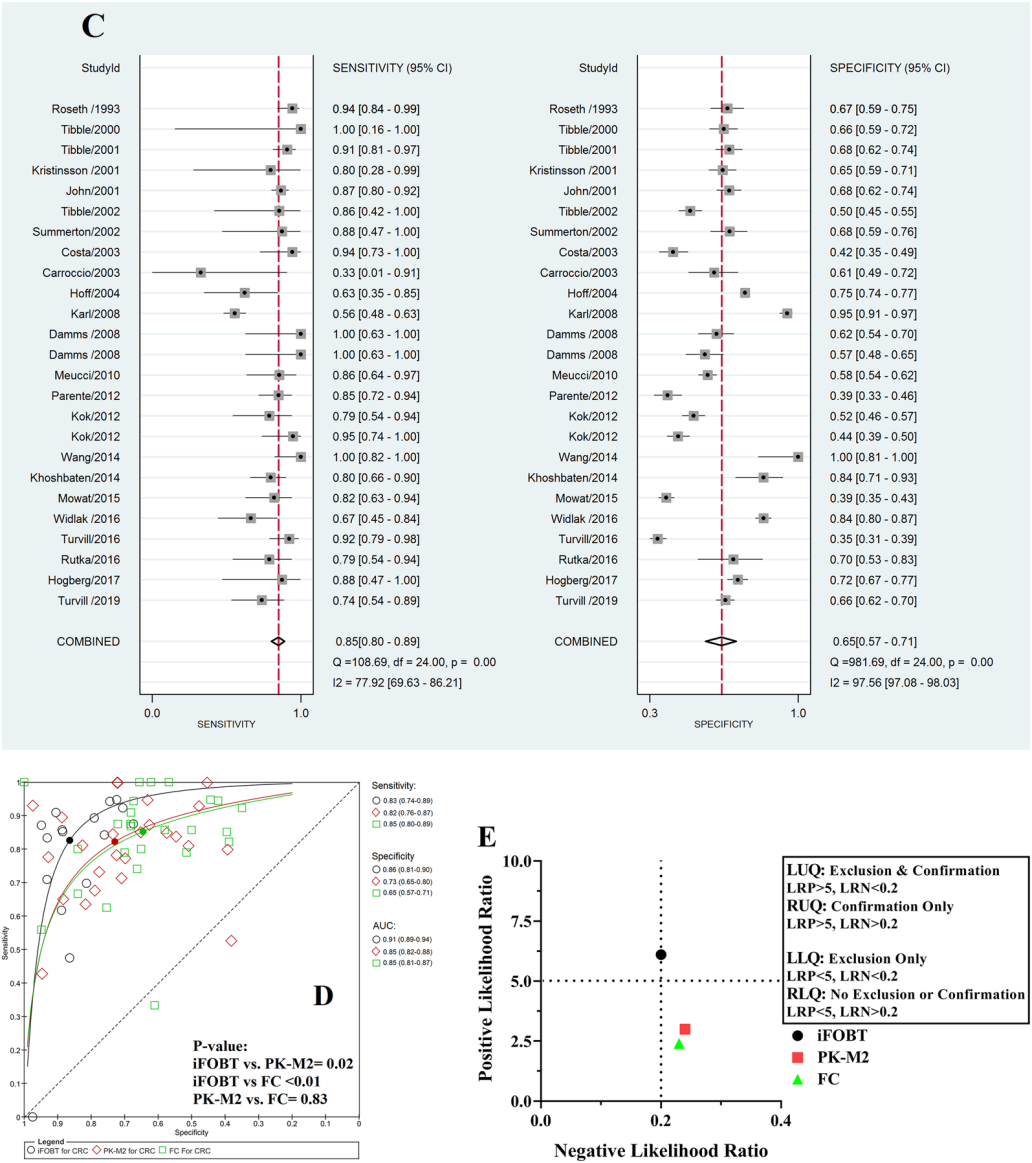
Forest plot, HSROC and LR scattergram of clinically applicable faecal protein biomarkers for CRC diagnosis. (**A**) Forest plot of iFOBT; (**B**) forest plot PK-M2; (**C**) forest plot of FC; (**D**) comparison of HSROCs of clinically applicable faecal protein biomarkers; (**E**) LR scattergram of clinically applicable faecal protein biomarkers. *CRC* colorectal cancer, *iFOBT* immunochemical faecal occult blood tests, *PK-M2* pyruvate kinase-M2, *FC* faecal calprotectin, *LUQ* left upper quadrant, *RUQ* right upper quadrant, *LLQ* left lower quadrant, *RLQ* right lower quadrant.

Table 2 presents the diagnostic accuracy of different faecal biomarkers for the detection of CRC, AA and AN. For CRC diagnosis, all 3 assessed biomarkers were applicable according to their PLR+, PLR− and AUC (> 2, < 0.5, and > 0.70, respectively) (Table 2 and Fig. 2A–D). Figure 2E shows the LR scattergram of CRC clinically applicable biomarkers.

**Figure 3:**
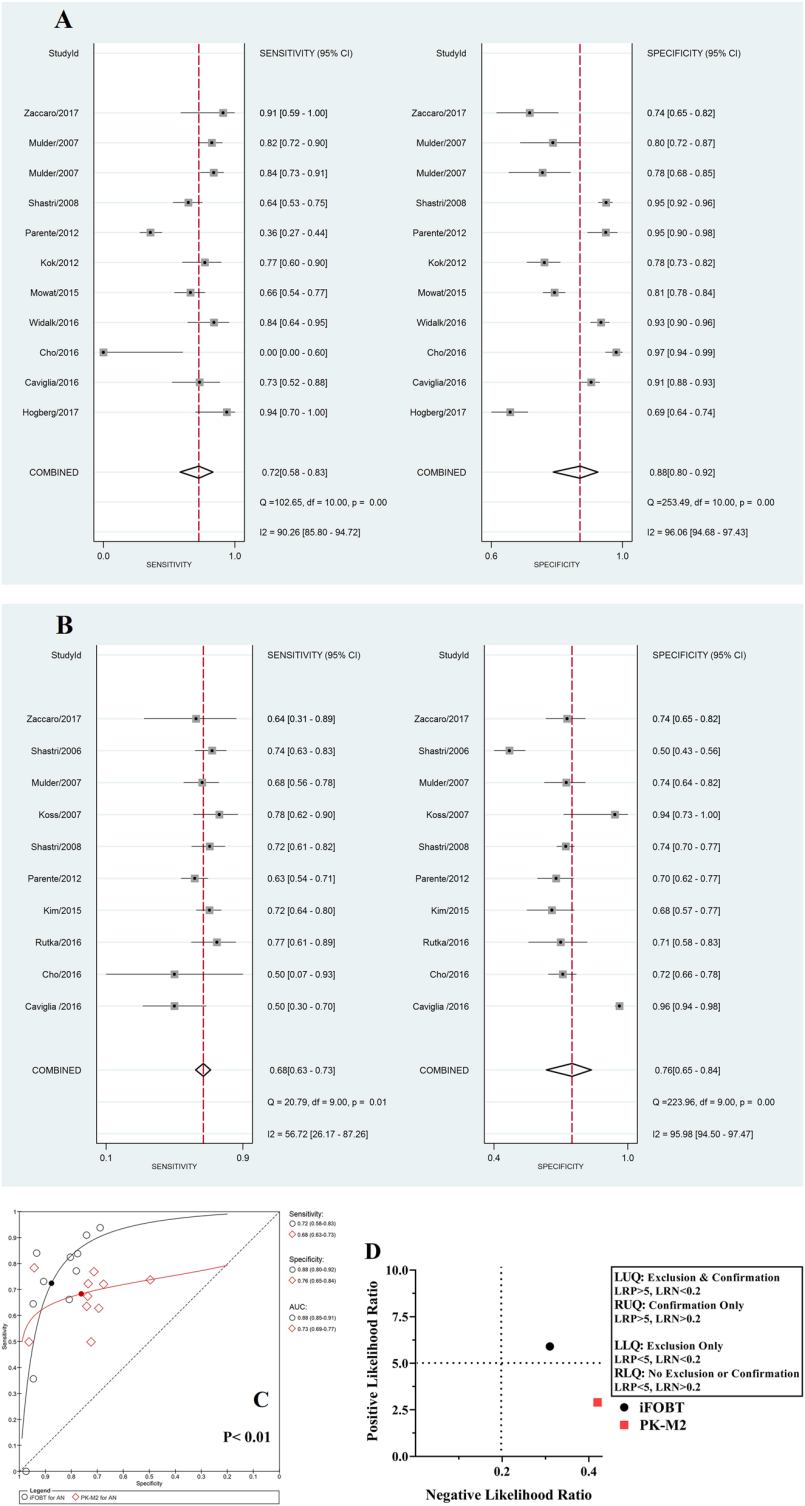
Forest plot, HSROC and LR scattergram of clinically applicable faecal protein biomarkers for AN diagnosis. (**A**) Forest plot of iFOBT; (**B**) Forest plot PK-M2; (**C**) comparison of the HSROCs of clinically applicable faecal protein biomarkers; (**D**) LR scattergram of clinically applicable faecal protein biomarkers. *AN* advanced neoplasms, *iFOBT* immunochemical faecal occult blood tests, *PK-M2* pyruvate kinase-M2, *LUQ* left upper quadrant, *RUQ* right upper quadrant, *LLQ* left lower quadrant, *RLQ* right lower quadrant.

Our results showed that there was no applicable biomarker for the diagnosis of AA individually. Moreover, the analyses showed that iFOBT and PK-M2 were clinically applicable for the detection of AN, whereas FC was not applicable (Table 2 and Fig. 3A–C). Figure 3D presents the LR scattergram of CRC clinically applicable biomarkers.

### Comparison of faecal biomarkers diagnostic accuracies

The most useful parameter for comparison of test accuracies between different biomarker groups or subgroups is DOR. Thus, we used individual DORs and their relatives to compare the diagnostic accuracies of clinically applicable biomarkers.

Among CRC clinically applicable biomarkers, the accuracy of iFOBT was significantly higher than that of PK-M2 and FC. The accuracies of PK-M2 and FC were not significantly different (Table 2). In addition, the AUC of iFOBT was the highest among other biomarkers, and based on the LR scattergram, only iFOBT had an upper moderate power of accuracy to both rule in and rule out CRC existence (Fig. 2E).

Among AN clinically applicable biomarkers, the accuracy of iFOBT was significantly higher than that of PK-M2. Furthermore, in comparison to PK-M2, the AUC of iFOBT was higher (Table 2). In line with the LR scattergram, iFOBT had an upper moderate power of accuracy to confirm but not exclude AN existence, whereas PK-M2 had a lower moderate power of accuracy to confirm and exclude AN existence (Fig. 3D).

To determine the effect of biomarker combinations on diagnostic accuracy, the results of double combinations, including iFOBT + PK-M2, iFOBT + FC, PK-M2 + FC, and triple combinations, namely, iFOBT + PK-M2 + FC, were extracted from primary studies if they had these data. The final result was considered positive if at least one of the biomarkers was positive, and negative results were determined if all double or triple combined biomarkers were negative. The iFOBT + PK-M2 data could be extracted from three studies^18,20,21^, following iFOBT + FC from three^20,23,25^, PK-M2 + FC from one^20^ and iFOBT + PK-M2 + FC from two^19,20^ studies. Our analysis could not find any combined biomarker that significantly increased the diagnostic accuracy compared to individual biomarkers (Supplemental Table S2). Moreover, PK-M2 + FC and iFOBT + PK-M2 + FC had significantly lower accuracy for the diagnosis of AN than individual iFOBTs.

### Subgroup analysis

Our results demonstrated substantial heterogeneity among studies in different groups when calculating the pooled sensitivity and specificity (Table 2). Thus, to identify the potential sources of heterogeneity, subgroup analyses were performed.

Each group of studies was separated into 7 subgroups on the basis of the method of measurements (latex agglutination immunoturbidimetry (LAIT) for iFOBT as well as enzyme-linked immunosorbent assay (ELISA) for PK-M2 and FC versus lateral flow), cut-off values (≥ 20 µg/g versus < 20 µg/g for iFOBT, > 4 U/mL versus 4 U/mL for PK-M2 and > 50 µg/g versus 50 µg/g for FC), study type (cohort versus case–control) and 4 domains of the QUADAS-2 “risk of bias” category (low risk versus high or unclear “risk of bias”) (Table 3). A significant difference in a subgroup indicates that it could be considered a source of heterogeneity.

**Table 3:** Subgroup analysis.

Test	Subgroup		No. of study	Sensitivity (95% CI)	Specificity (95% CI)	RDOR (95% CI)	P value
**Colorectal cancer**							
iFOBT	Method of measurement	LAIT	6	0.77 (0.60–0.93)	0.90 (0.86–0.95)	1.76 (0.65–4.80)	0.24
		LF	9	0.84 (0.75–0.94)	0.81 (0.74–0.88)		
	Cut off	≥ 20 µg/g	5	0.86 (0.73–0.98)	0.83 (0.74–0.92)	0.84 (0.24–2.96)	0.77
		< 20 µg/g	11	0.81 (0.72–0.91)	0.88 (0.83–0.93)		
	Study type	C	12	0.85 (0.77–0.93)	0.85 (0.80–0.91)	1.01 (0.28–3.65)	0.98
		CC	4	0.76 (0.59–0.92)	0.90 (0.83–0.97)		
	Patient selection	Low	12	0.85 (0.77–0.93)	0.85 (0.80–0.91)	0.99 (0.27–3.57)	0.98
		High/unclear	4	0.76 (0.59–0.92)	0.90 (0.83–0.97)		
	Index test	Low	11	0.83 (0.74–0.92)	0.86 (0.81–0.92)	0.96 (0.29–3.15)	0.94
		High/unclear	5	0.81 (0.67–0.95)	0.87 (0.79–0.95)		
	Reference standard	Low	11	0.81 (0.71–0.91)	0.89 (0.85–0.93)	1.85 (0.57–6.07)	0.28
		High/unclear	5	0.86 (0.74–0.97)	0.79 (0.69–0.89)		
	Flow and timing	Low	10	0.83 (0.74–0.93)	0.86 (0.80–0.92)	0.95 (0.30–2.97)	0.92
		High/unclear	6	0.82 (0.69–0.94)	0.87 (0.80–0.94)		
PK-M2	Method of measurement	ELISA	20	0.78 (0.73–0.83)	0.72 (0.63–0.81)	0.14 (0.04–0.48)	< 0.01
		LF	5	0.95 (0.90–0.99)	0.76 (0.61–0.91)		
	Cut off	> 4 U/mL	2	0.70 (0.46–0.93)	0.64 (0.31–0.97)	3.71 (0.56–24.49)	0.16
		4 U/mL	23	0.83 (0.78–0.88)	0.73 (0.66–0.81)		
	Study type	C	11	0.86 (0.79–0.93)	0.70 (0.58–0.82)	0.86 (0.27–2.66)	0.77
		CC	14	0.79 (0.72–0.86)	0.75 (0.66–0.85)		
	Patient selection	Low	11	0.86 (0.79–0.93)	0.70 (0.58–0.82)	1.17 (0.38–3.64)	0.77
		High/unclear	14	0.79 (0.72–0.86)	0.75 (0.66–0.85)		
	Index test	Low	14	0.87 (0.82–0.91)	0.71 (0.61–0.81)	1.68 (0.55–5.09)	0.34
		High/Unclear	11	0.75 (0.68–0.83)	0.75 (0.64–0.87)		
	Reference standard	Low	20	0.82 (0.76–0.88)	0.75 (0.68–0.83)	1.66 (0.44–6.32)	0.43
		High/Unclear	5	0.83 (0.73–0.94)	0.62 (0.42–0.81)		
	Flow and timing	Low	19	0.80 (0.74–0.86)	0.74 (0.66–0.83)	0.79 (0.21–3.07)	0.72
		High/Unclear	6	0.88 (0.81–0.96)	0.69 (0.51–0.86)		
FC	Method of measurement	ELISA	23	0.85 (0.81–0.89)	0.65 (0.58–0.72)	1.64 (0.37–7.32)	0.49
		LF	2	0.86 (0.70–1.00)	0.57 (0.31–0.83)		
	Cut off	> 50 μg/g	4	0.83 (0.72–0.94)	0.78 (0.65–0.92)	1.22 (0.44–3.37)	0.68
		50 μg/g	21	0.85 (0.81–0.90)	0.62 (0.55–0.69)		
	Study type	C	18	0.84 (0.78–0.90)	0.61 (0.52–0.69)	3.47 (1.58–7.60)	0.03
		CC	7	0.90 (0.83–0.97)	0.74 (0.63–0.85)		
	Patient selection	Low	18	0.84 (0.78–0.90)	0.61 (0.52–0.69)	0.29 (0.13–0.63)	0.03
		High/unclear	7	0.90 (0.83–0.97)	0.74 (0.63–0.85)		
	Index test	Low	15	0.86 (0.80–0.91)	0.60 (0.51–0.69)	0.65 (0.30–1.40)	0.25
		High/Unclear	10	0.84 (0.78–0.91)	0.72 (0.62–0.81)		
	Reference standard	Low	24	0.85 (0.81–0.89)	0.64 (0.57–0.71)	0.53 (0.04–6.64)	0.60
		High/Unclear	1	0.88 (0.63–1.00)	0.72 (0.42–1.00)		
	Flow and timing	Low	13	0.88 (0.83–0.93)	0.64 (0.54–0.74)	1.48 (0.72–3.04)	0.26
		High/Unclear	12	0.83 (0.77–0.89)	0.65 (0.55–0.75)		
**Advanced adenoma**							
iFOBT	Method of measurement	LAIT	5	0.40 (0.19–0.61)	0.88 (0.82–0.95)	1.50 (0.14–16.54)	0.69
		LF	5	0.66 (0.48–0.83)	0.70 (0.56–0.84)		
	Cut off	≥ 20 µg/g	4	0.60 (0.33–0.87)	0.75 (0.58–0.92)	0.87 (0.12–6.36)	0.87
		< 20 µg/g	6	0.48 (0.23–0.72)	0.84 (0.75–0.94)		
	Index test	Low	8	0.53 (0.34–0.72)	0.83 (0.73–0.92)	2.08 (0.20–21.12)	0.47
		High/Unclear	2	0.50 (0.10–0.90)	0.73 (0.47–0.98)		
	Reference Standard	Low	7	0.47 (0.28–0.65)	0.87 (0.81–0.92)	4.58 (0.39–54.43)	0.18
		High/Unclear	3	0.71 (0.49–0.94)	0.60 (0.43–0.77)		
	Flow and Timing	Low	7	0.46 (0.24–0.68)	0.81 (0.70–0.92)	0.45 (0.08–2.63)	0.31
		High/Unclear	3	0.62 (0.35–0.89)	0.81 (0.65–0.98)		
PK-M2	Method of measurement	ELISA	7	0.39 (0.27–0.50)	0.68 (0.52–0.85)	1.72 (0.13–22.98)	0.63
		LF	3	0.64 (0.46–0.82)	0.53 (0.24–0.81)		
	Study type	C	8	0.40 (0.29–0.51)	0.38 (0.08–0.68)	2.31 (0.07–74.63)	0.58
		CC	2	0.70 (0.49–0.90)	0.70 (0.56–0.83)		
	Patient selection	Low	8	0.40 (0.29–0.51)	0.70 (0.56–0.83)	0.43 (0.01–14.02)	0.58
		High/Unclear	2	0.70 (0.49–0.90)	0.38 (0.08–0.68)		
	Index test	Low	8	0.44 (0.30–0.58)	0.67 (0.51–0.83)	0.90 (0.06–13.03)	0.92
		High/Unclear	2	0.53 (0.24–0.81)	0.51 (0.15–0.87)		
	Reference standard	Low	8	0.44 (0.30–0.58)	0.69 (0.55–0.83)	1.83 (0.13–24.82)	0.60
		High/Unclear	2	0.52 (0.27–0.78)	0.42 (0.10–0.75)		
	Flow and timing	Low	8	0.40 (0.29–0.51)	0.61 (0.44–0.78)	0.20 (0.03–1.33)	0.08
		High/Unclear	2	0.62 (0.42–0.81)	0.76 (0.50–1.00)		
FC	Method of measurement	ELISA	9	0.43 (0.33–0.53)	0.56 (0.45–0.68)	0.41 (0.10–1.58)	0.16
		LF	1	0.69 (0.36–1.00)	0.51 (0.16–0.85)		
	Study type	C	9	0.42 (0.33–0.52)	0.40 (0.08–0.72)	3.84 (0.18–81.07)	0.33
		CC	1	0.88 (0.62–1.00)	0.58 (0.47–0.68)		
	Patient selection	Low	9	0.42 (0.33–0.52)	0.58 (0.47–0.68)	0.26 (0.01–5.50)	0.33
		High/Unclear	1	0.88 (0.62–1.00)	0.40 (0.08–0.72)		
	Index test	Low	7	0.45 (0.28–0.62)	0.59 (0.47–0.71)	1.11 (0.26–4.71)	0.87
		High/Unclear	3	0.56 (0.29–0.83)	0.48 (0.29–0.68)		
	Reference standard	Low	9	0.46 (0.35–0.56)	0.54 (0.43–0.65)	0.41 (0.04–3.77)	0.37
		High/Unclear	1	0.50 (0.06–0.94)	0.71 (0.44–0.98)		
	Flow and timing	Low	5	0.39 (0.22–0.56)	0.57 (0.42–0.72)	0.62 (0.30–1.24)	0.14
		High/Unclear	5	0.48 (0.32–0.64)	0.55 (0.39–0.70)		
**Advanced neoplasm**							
iFOBT	Method of measurement	LAIT	6	0.61 (0.44–0.79)	0.91 (0.86–0.97)	1.07 (0.34–3.33)	0.89
		LF	5	0.82 (0.70–0.93)	0.82 (0.72–0.93)		
	Cut off	≥ 20 µg/g	5	0.77 (0.60–0.94)	0.84 (0.74–0.94)	1.65 (0.58–4.65)	0.29
		< 20 µg/g	6	0.68 (0.49–0.86)	0.90 (0.84–0.96)		
	index test	Low	8	0.71 (0.57–0.85)	0.89 (0.83–0.95)	1.17 (0.28–4.81)	0.80
		High/Unclear	3	0.74 0.49–0.99)	0.83 (0.69–0.97)		
	Reference Standard	Low	7	0.61 (0.48–0.74)	0.92 (0.88–0.95)	1.13 (0.22–5.74)	0.86
		High/Unclear	4	0.87 (0.78–0.96)	0.76 (0.64–0.87)		
	Flow and Timing	Low	8	0.73 (0.58–0.88)	0.89 (0.83–0.95)	1.83(0.66–5.04)	0.20
		High/Unclear	3	0.71 (0.47–0.96)	0.84 (0.70–0.97)		
PK-M2	Method of measurement	ELISA	7	0.67 (0.61–0.72)	0.78 (0.68–0.89)	1.28 (0.62–2.65)	0.44
		LF	3	0.72 (0.65–0.80)	0.71 (0.51–0.90)		
	Study type	C	8	0.67 (0.61–0.72)	0.76 (0.65–0.86)	1.40 (0.60–3.28)	0.38
		CC	2	0.72 (0.64–0.81)	0.80 (0.59–1.00)		
	Patient selection	Low	8	0.67 (0.61–0.72)	0.76 (0.65–0.86)	0.72 (0.30–1.68)	0.38
		High/Unclear	2	0.72 (0.64–0.81)	0.80 (0.58–1.00)		
	Index test	Low	7	0.70 (0.66–0.74)	0.76 (0.74–0.78)	1.55 (0.82–2.92)	0.15
		High/Unclear	3	0.66 (0.58–0.73)	0.73 (0.67–0.78)		
	Reference standard	Low	7	0.68 (0.62–0.73)	0.78 (0.67–0.89)	0.98 (0.49–1.95)	0.93
		High/Unclear	3	0.70 (0.62–0.77)	0.72 (0.53–0.91)		
	Flow and timing	Low	8	0.69 (0.64–0.74)	0.72 (0.62–0.81)	0.85 (0.38–1.93)	0.66
		High/Unclear	2	0.64 (0.53–0.75)	0.88 (0.78–0.98)		
FC	Method of measurement	ELISA	9	0.70 (0.58–0.81)	0.60 (0.49–0.71)	1.14 (0.10–12.94)	0.89
		LF	1	0.75 (0.44–1.00)	0.52 (0.18–0.87)		
	Study type	C	9	0.67 (0.56–0.78)	0.61 (0.51–0.72)	3.21 (0.14–74.04)	0.40
		CC	1	0.93 (0.80–1.00)	0.43 (0.10–0.76)		
	Patient selection	Low	9	0.67 (0.56–0.78)	0.61 (0.51–0.72)	0.31 (0.01–7.20)	0.40
		High/Unclear	1	0.93 (0.80–1.00)	0.43 (0.10–0.76)		
	Index test	Low	7	0.67 (0.54–0.81)	0.63 (0.51–0.74)	1.08 (0.19–6.09)	0.91
		High/Unclear	3	0.76 (0.59–0.94)	0.52 (0.32–0.71)		
	Reference standard	Low	9	0.70 (0.59–0.82)	0.58 (0.47–0.69)	0.53 (0.04–6.74)	0.57
		High/Unclear	1	0.69 (0.31–1.00)	0.73 (0.46–0.99)		
	Flow and timing	Low	5	0.74 (0.61–0.88)	0.63 (0.49–0.77)	2.07 (0.55–7.75)	0.23
		High/Unclear	5	0.64 (0.48–0.81)	0.56 (0.41–0.71)		

*iFOB* immunochemical faecal occult blood tests, *PK-M2* pyruvate kinase-M2, *FC* faecal calprotectin, *LAIT* latex agglutination immunoturbidimetry, *LF* lateral flow, *C* cohort study design, *CC* case–control study design.

**Figure 4:**
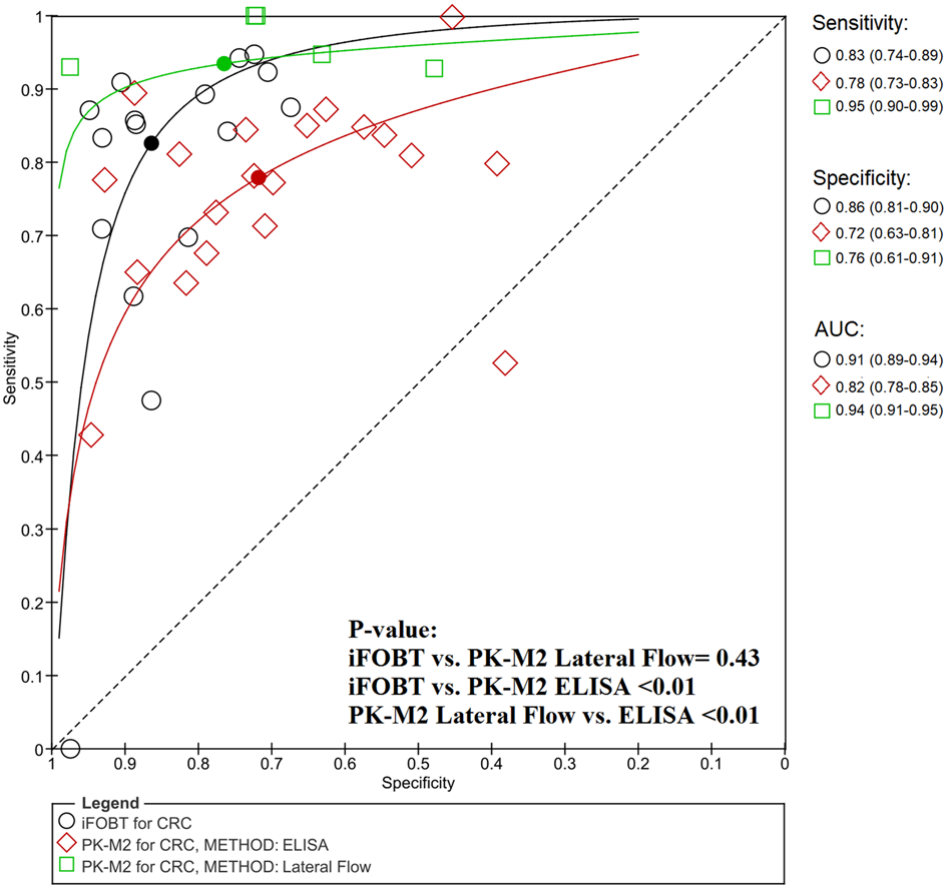
Comparison of the HSROCs of PK-M2 with different methods of measurement. *iFOBT* immunochemical faecal occult blood tests, *PK-M2* pyruvate kinase-M2, *ELISA* enzyme-linked immunosorbent assay.

For the diagnosis of CRC, the lateral flow method of PK-M2 measurement led to a significant increase in the overall accuracy (Fig. 4). Moreover, in the FC group, the case–control study design and high or unclear “risk of bias” in the “patient selection” domain led to a significant increase in overall accuracy.

Regarding the detection of AA and AN, there was no subgroup to change the overall accuracy. Due to the similar subset of each covariate, subgroup analyses of study type and “patient selection” domain in iFOBT and cut-off value in PK-M2 and FC groups were not feasible for AA diagnosis. Additionally, the study type and “patient selection” domain in iFOBT and the cut-off value in the PK-M2 and FC groups were not executable in the AN group.

### Threshold effect and meta-regression analysis

In addition to subgroup analysis, threshold effect and univariate meta-regression analysis were performed to further evaluate causes of heterogeneity.

In diagnostic accuracy studies, one of the most important sources of heterogeneity is the threshold effect. Our analysis showed that the diagnostic threshold effect was not significant as a source of heterogeneity for iFOBT and FC to CRC, AA, and AN diagnosis. Regarding PK-M2, although there was no significant threshold effect in the CRC and AA groups, there was significant heterogeneity in AN detection (P < 0.01) (Table 2).

For univariate meta-regression analysis, we considered some covariates, including the mean age of patients, % male as sex frequency, % distal tumours as CRC tumour site, and % late as CRC tumour stage. Our results demonstrated that none of the aforementioned covariates had sensitivity and specificity heterogeneity. It should be noted that due to the lack of FC biomarker data, analysis of the aforementioned covariates in the AA group as well as the impact of CRC tumour stage on heterogeneity were not feasible (Table 4).

**Table 4:** Univariate meta-regression.

Test		Covariate	No. of study	Sensitivity (95% CI)	P value	Specificity (95% CI)	P value
iFOBT	CRC	Age	16	0.84 (0.75–0.90)	0.89	0.86 (0.81–0.90)	0.89
		%Male	16	0.81 (0.73–0.88)	0.78	0.87 (0.83–0.91)	0.85
		%Distal	6	0.90 (0.81–0.95)	0.95	0.86 (0.76–0.92)	0.95
		%Late	6	0.86 (0.70–0.94)	0.99	0.85 (0.77–0.90)	0.95
	AA	Age	10	0.54 (0.36–0.72)	0.90	0.80 (0.70–0.88)	0.89
		%Male	10	0.52 (0.35–0.68)	0.89	0.82 (0.73–0.88)	0.87
	AN	Age	11	0.73 (0.59–0.84)	0.90	0.87 (0.80–0.92)	0.88
		%Male	11	0.72 (0.61–0.81)	0.90	0.88 (0.82–0.92)	0.92
PK-M2	CRC	Age	22	0.83 (0.77–0.87)	0.93	0.74 (0.65–0.82)	0.83
		%Male	22	0.82 (0.76–0.87)	0.98	0.73 (0.63–0.81)	1.00
		%Distal	8	0.87 (0.75–0.94)	0.99	0.80 (0.68–0.89)	0.92
		%Late	10	0.81 (0.74–0.87)	1.00	0.71 (0.64–0.78)	0.99
	AA	Age	10	0.45 (0.34–0.58)	0.91	0.64 (0.48–0.78)	0.99
		%Male	10	0.47 (0.35–0.60)	0.97	0.65 (0.49–0.78)	0.95
	AN	Age	10	0.68 (0.64–0.73)	0.90	0.77 (0.67–0.85)	0.89
		%Male	10	0.68 (0.64–0.73)	0.97	0.77 (0.66–0.84)	0.97
FC	CRC	Age	22	0.85 (0.79–0.89)	0.92	0.65 (0.57–0.73)	0.97
		%Male	21	0.83 (0.77–0.87)	0.96	0.65 (0.57–0.73)	0.93
		%Distal	4	0.88 (0.71–0.95)	0.91	0.80 (0.59–0.92)	0.97
	AN	Age	9	0.69 (0.55–0.80)	0.89	0.59 (0.47–0.70)	0.93
		%Male	9	0.69 (0.56–0.80)	0.93	0.57 (0.46–0.68)	0.91

*iFOB* immunochemical faecal occult blood tests, *PK-M2* pyruvate kinase-M2, *FC* faecal calprotectin, *CRC* colorectal cancer, *AA* advanced adenoma, *AN* advanced neoplasms.

## Discussion

For the first time, our present systematic review and meta-analysis summarized and compared the diagnostic performances of all available faecal protein biomarkers, namely, iFOBT, PK-M2 and FC, for screening CRC, AA, and AN. Additionally, and uniquely, we assessed the impact of tumour site, tumour stage, method of measurement and different cut-off values on the performance of these biomarkers.

The overall quality of the included studies for each biomarker was relatively high according to the QUADAS-2 tool. In summary, the range of low-risk studies in the “risk of bias” category for all four domains was 50–95.6%, reflecting moderate to very low risk of bias, and all included studies had no concern regarding “applicability” in all three domains. To evaluate the impact of QUADAS-2 domains on the overall accuracy, subgroup analysis was conducted based on low versus high or unclear risk from the “risk of bias” category. The results showed that despite the impact of different domains on sensitivities and specificities, only the “patient selection” domain in the FC group for CRC detection could significantly affect the overall accuracy, which we have discussed in the fourth following paragraph (Table 3).

The first important aim of our study was to determine the most accurate faecal protein biomarker. Our analyses showed that iFOBT, PK-M2 and FC biomarkers were clinically applicable for CRC, as well as iFOBT and PK-M2 for AN, and there were no biomarkers for AA according to their AUCs or positive and negative LRs. Additionally, the combination of biomarkers could not increase the accuracy for the detection of each condition. The overall accuracy of iFOBT was significantly higher than that of PK-M2 and FC for CRC detection (P = 0.02 and < 0.01, respectively) and significantly higher than that of PK-M2 for AN diagnosis (P < 0.01). Pursuant to our search results, before ours, there was no meta-analysis to compare the accuracy of various faecal biomarkers for the diagnosis of different intestinal neoplasms. Nonetheless, Li et al*.*^56^, using 4 research papers, conducted a direct comparison between iFOBT and PK-M2 for CRC screening. Despite the small number of studies to achieve convincing results, to confirm our findings, they indicated that iFOBT had significantly higher accuracy than PK-M2. Furthermore, all of our included articles that contained comparison data had higher iFOBT accuracy than PK-M2 and/or FC for the diagnosis of both CRC and AN, except the results of Kim et al*.*^13^. Kim et al*.* assessed the accuracy of two different methods of PK-M2 measurement and compared them with iFOBT in CRC and adenoma patients. Their results showed that regardless of the measurement method, PK-M2 accuracy was superior to iFOBT for the diagnosis of CRC and adenoma. The most likely reason for this contradiction is a technical mistake related to measurement equipment, in which a systematic error gave rise to a decrease in the accuracy of iFOBT in Kim’s study. To clarify this issue, the iFOBT accuracy of Kim’s study was tested versus the other studies. The results indicated that the performance of iFOBT in Kim’s study was significantly lower than that in other studies (RDOR = 0.19 (95% CI, 0.04–0.10); P = 0.04), which indicates that systematic error is possible.

Today, the most widely used biomarker for the detection of colorectal neoplasms is FOBT. Two commonly used FOBTs are gFOBT and iFOBT, and it has been proven that iFOBT has superior diagnostic performance^2,57^. Our results showed that iFOBT is clinically applicable for CRC diagnosis with upper moderate overall accuracy, in line with its positive and negative LR results. Additionally, it is clinically applicable for AN with upper moderate accuracy only for confirmation, not for exclusion. The overall accuracy of iFOBT in our present study is similar to that of previously published meta-analyses^58–60^. However, we evaluated more covariates in our research to shed light on the different strengths and limitations of iFOBT implementation. The first unique covariate was the measurement method. Currently, there are two common methods for the measurement of iFOBT, qualitative rapid lateral flow and quantitative latex agglutination immunoturbidimetry, whereas before the present study, there were no data about their overall accuracy differences. According to our findings, there was no difference between these two methods of measurement with different commercial brands for the diagnosis of all three conditions (Table 3). Another covariate was the cut-off to find the optimal iFOBT value. In a previously published meta-analysis, Lee et al.^58^ proposed that a lower 20 μg/g cut-off may increase the sensitivity of iFOBT for the detection of CRC compared to the upper 20 μg/g values. Therefore, we analysed the difference in accuracies between the lower 20 μg/g versus upper 20 μg/g values not only for CRC detection but also for AA and AN. Our results indicated that there were no significant differences among different cut-off values for the detection of CRC, AA and AN (Table 3). Meanwhile, the results of univariate meta-regression analysis showed that age, sex, CRC tumour site and stage could not affect the sensitivity and specificity of iFOBT for the diagnosis of all three conditions (Table 4). The results of the most recently published meta-analysis confirmed our findings in terms of the impact of tumour site on iFOBT performance^60^. However, concerning CRC tumour site, the results of Hirai et al*.’s* meta-analysis^59^ are not completely consistent with ours. They concluded that the overall accuracy of iFOBT for the proximal colon was significantly lower than that for the distal colon, but it is not convincing given the largely overlapping confidence intervals in the site-specific sensitivities.

PK-M2 is a promising non-organ-specific tumour biomarker, and its concentration is elevated in various types of tumours^56^. For the first time in 2004, Hardt et al.^41^ demonstrated that the PK-M2 concentration was elevated in the faeces of CRC patients and could be used as a biomarker. To date, several studies have been conducted on faecal PK-M2 in CRC patients, and the results have shown contradictory accuracies. To determine whether faecal PKM2 could be used as a biomarker for the diagnosis of colorectal neoplasms, a diagnostic accuracy meta-analysis must be carried out. Following two earlier versions^56,61^, the latest diagnostic accuracy meta-analysis of PK-M2 for CRC detection was published in 2015, which included 8 studies^6^. Nonetheless, all aforementioned studies included only CRC patients, without evaluating the impact of different covariates on PK-M2 performance. In this study, plus updating the body of evidence using 26 included research articles, we uniquely assessed the diagnostic accuracy of PK-M2 for the detection of AA and AN in addition to CRC. Furthermore, the impact of different covariates on the performance of PK-M2 was evaluated. Our findings indicated that PK-M2 was clinically applicable for the diagnosis of CRC and AN and not for AA, with lower moderate accuracy for both disease confirmation and exclusion given its LR results. These results are compatible with previous meta-analyses regarding the accuracy of PK-M2 for the diagnosis of CRC^6,56,61^. To provide new insights into PK-M2 performance, we assessed different covariates in terms of accuracy. One of our important findings was the impact of the PK-M2 measurement method on its performance. Subgroup analysis in the CRC group demonstrated that rapid lateral flow could significantly increase the accuracy of PK-M2 compared to the ELISA method (RDOR = 0.14 (95% CI 0.04–0.48); P < 0.01) (Fig. 4). These findings were similar to the study results of Kim et al*.*^13^. Moreover, we reanalysed the difference in iFOBT and lateral flow PK-M2 measurement accuracies. The results revealed that when lateral flow PK-M2 measurement was implemented, it eliminated the initial significant difference in iFOBT accuracy for CRC detection (RDOR = 1.79 (95% CI 0.38–8.46); P = 0.43), whereas the accuracy of iFOBT for AN was still significantly superior to that of lateral flow PK-M2 (RDOR = 0.28 (95% CI 0.10–0.81); P = 0.02). The lower accuracy of the ELISA method could be derived from the biostability of tumour PK-M2 in stool samples. There is some evidence that tumour PK-M2 in stool samples could be dramatically affected by sample storage time^62^. By nature, ELISA is a time-consuming method, whereas lateral flow is a rapid technique that is commonly utilized in point-of-care tests (POCTs). Additionally, our results implied that age, sex, cut-off value, CRC tumour site and stage did not affect PK-M2 accuracy (Tables 3, 4).

FC is released in faeces following mucosal neutrophil degradation as a result of intestinal inflammation. The level of FC increases in a wide range of intestinal diseases that are associated with inflammation, including inflammatory bowel disease, CRC and AA^55^. The results of numerous studies indicated a broad range of FC sensitivities for the detection of CRC, from 33 to 100% (Fig. 2C). The latest meta-analysis with 20 included articles regarding the performance of FC for CRC and adenoma diagnosis was performed in 2018^7^. However, this prior paper evaluated all adenomas, not advanced type adenomas, which are clinically important precursors of CRC. Meanwhile, there were no data concerning the impact of measurement technique, type of included studies, and CRC site specificity on FC accuracy. In the present research, in addition to updating the data using 23 included research articles regarding CRC, we assessed the diagnostic accuracy of FC in AA and AN detection for the first time as well as the impact of various covariates on FC performance. Our results are consistent with a previous meta-analysis^7^ indicating that FC has lower moderate accuracy for the diagnosis of CRC based on its LR values. Additionally, we determined that it is not applicable to the detection of AA and AN. Evaluated covariates, including age, sex, method of measurement and CRC tumour site, had no significant effect on FC accuracy (Table 4). Nonetheless, the case–control study design and “patient selection’ domain from the QUADAS-2 “risk of bias” category had a significant impact on FC performance for the diagnosis of CRC (Table 3). These two covariates are relatively similar because a high-risk point is given to case–control studies in the “patient selection’ domain. As mentioned above, FC has low specificity for intestinal disorders; therefore, its overall accuracy declines in cohort study designs that include patients with different intestinal disorders.

One of the most important strengths of this study was the adoption of rigorous inclusion and exclusion criteria in three widely used medical databases without language restriction. Diagnostic accuracy comparison of multiple biomarkers and subgroup analysis by different methods of measurement and cut-off values are another unique strength. In addition, we analysed the impact of the site and the stage of tumours on the biomarker performances in the CRC group, which has not been conducted in previous meta-analyses. Despite the strengths, there are some limitations that should be taken into consideration when interpreting our findings. First, the accuracy of AN detection may be under- or overestimated because it is strongly influenced by the proportion of CRC and AA cases in the study population. Second, AA data were not available to determine site-specific accuracy. Third, the protocol of this study has not been registered on the PROSPERO database.

## Conclusion

In summary, our results determined that iFOBT is the most accurate faecal biomarker and is recommended for the diagnosis of CRC and AN, among other clinically applicable types. In addition, the lateral flow method of PK-M2 measurement should be implemented instead of ELISA due to its higher efficacy on PK-M2 performance. There is no clinically applicable faecal biomarker for AA diagnosis as an important precursor of CRC. Large prospective cohort studies are recommended to confirm our findings. Additionally, further research is suggested to find new comprehensive biomarkers.

## Supplementary Information

Supplementary Information.
