# Supplementary material for: Comparison of faecal protein biomarkers' diagnostic accuracy for colorectal advanced neoplasms: a systematic review and meta-analysis
Source: Sci Rep. 2022 Feb 16;12:2623. doi: 10.1038/s41598-022-06689-4 (PMC8850428; doi:10.1038/s41598-022-06689-4)
Supplement: Supplementary file 1 — Supplementary Information. [file 41598_2022_6689_MOESM1_ESM.pdf]

# Comparison of faecal protein biomarkers diagnostic accuracy for colorectal advanced neoplasms: a systematic review and meta-analysis

**Atefeh Nasir Kansestani[1•], Mohammad Erfan Zare[1•], Qingchao Tong[1], Jun Zhang[1*]**

[1] Department of Clinical Laboratory, Sir Run Run Shaw Hospital, Zhejiang University, School of Medicine, Hangzhou, China

• These two authors work equally.

* **Correspondence:** Professor Jun Zhang, Department of Clinical Laboratory, Sir Run Run Shaw Hospital, Zhejiang University, School of Medicine, Hangzhou, China. Tel: (+86 571) 8600 2260, Email:jameszhang2000@zju.edu.cn

**Supplemental Table 1.** Strategies for database search.

| Database | Search Strategy |
|---|---|
| **PubMed**<br>http://www.ncbi.nlm.nih.gov/pubmed | **MeSH terms:** ("Colorectal neoplasms") AND ("Diagnosis" OR "Early detection of cancer")<br>**Text words:** ("Colorectal cancer" OR "CRC" OR "Colorectal malignancy" OR "Colorectal tumour" OR "Adenoma" OR "Colorectal neoplasms") AND ("Faecal biomarker" OR "Laboratory tests" "Diagnostic biomarker" OR "Screening Biomarker") |
| **Web of Science**<br>http://apps.webofnowledge.com/ | **Text words:** ("Colorectal cancer" OR "CRC" OR "Colorectal malignancy" OR "Colorectal tumour" OR "Adenoma" OR "Colorectal neoplasms") AND ("Faecal biomarker" OR "Laboratory tests" "Diagnostic biomarker" OR "Screening Biomarker") |
| **Scopus**<br>http://www.scopus.com/ | **Text words:** ("Colorectal cancer" OR "CRC" OR "Colorectal malignancy" OR "Colorectal tumour" OR "Adenoma" OR "Colorectal neoplasms") AND ("Faecal biomarker" OR "Laboratory tests" "Diagnostic biomarker" OR "Screening Biomarker") |

**Supplemental Table 2.** Relative-diagnostic Odds Ratio of combined biomarkers compared to individual biomarkers

| Biomarker | iFOBT+PK-M2 | iFOBT+FC | PK-M2+FC | iFOBT+PK-M2+FC |
|---|---|---|---|---|
| **Colorectal Cancer** | | | | |
| **iFOBT** | 0.54 (0.07-4.02); P= 0.51 | 0.57 (0.10-2.86); P= 0.46 | 0.12 (0.01-2.30); P= 0.14 | 0.11 (0.01-2.07); P= 0.12 |
| **PK-M2** | 2.49 (0.27-22.87); P= 0.40 | 1.71 (0.33-8.91); P= 0.51 | 0.95 (0.04-21.56); P= 0.97 | 0.95 (0.04-22.11); P= 0.97 |
| **FC** | 2.25 (0.53-9.56); P= 0.26 | 1.75 (0.58-5.27); P= 0.30 | 0.90 (0.12-7.05); P= 0.91 | 1.00 (0.13-7.90); P= 0.99 |
| **Advance Adenoma** | | | | |
| **iFOBT** | 1.14 (0.06-21.17); P= 0.92 | 1.47 (0.11-20.34); P= 0.75 | 0.13 (0.01-3.40); P= 0.18 | 0.15 (0.01-3.16); P= 0.19 |
| **PK-M2** | 7.91 (0.33-188.14); P= 0.17 | 4.92 (0.33-72.93); P=0.21 | 0.72 (0.02-22.43); P= 0.83 | 0.52 (0.02-14.30); P= 0.66 |
| **FC** | 3.25 (0.42-24.93); P= 0.22 | 1.61 (0.28-9.27); P= 0.55 | 0.56 (0.10-3.01); P= 0.44 | 1.93 (0.33-11.42); P= 0.42 |
| **Advance Adenoma** | | | | |
| **iFOBT** | 0.43 (0.08-2.24); P= 0.28 | 0.59 (0.09-3.76); P= 0.54 | 0.07 (0.01-0.43); P< 0.01 | 0.05 (0.01-0.33); P< 0.01 |
| **PK-M2** | 1.31 (0.58-2.97); P= 0.46 | 3.45 (0.81-14.67); P= 0.86 | 0.86 (0.28-2.66); P= 0.77 | 0.85 (0.29-2.47); P= 0.73 |
| **FC** | 4.00 (0.51-31.68); P= 0.16 | 3.68 (0.57-23.74); P= 0.15 | 0.45 (0.04-4.68); P= 0.45 | 0.36 (0.04-3.58); P= 0.33 |

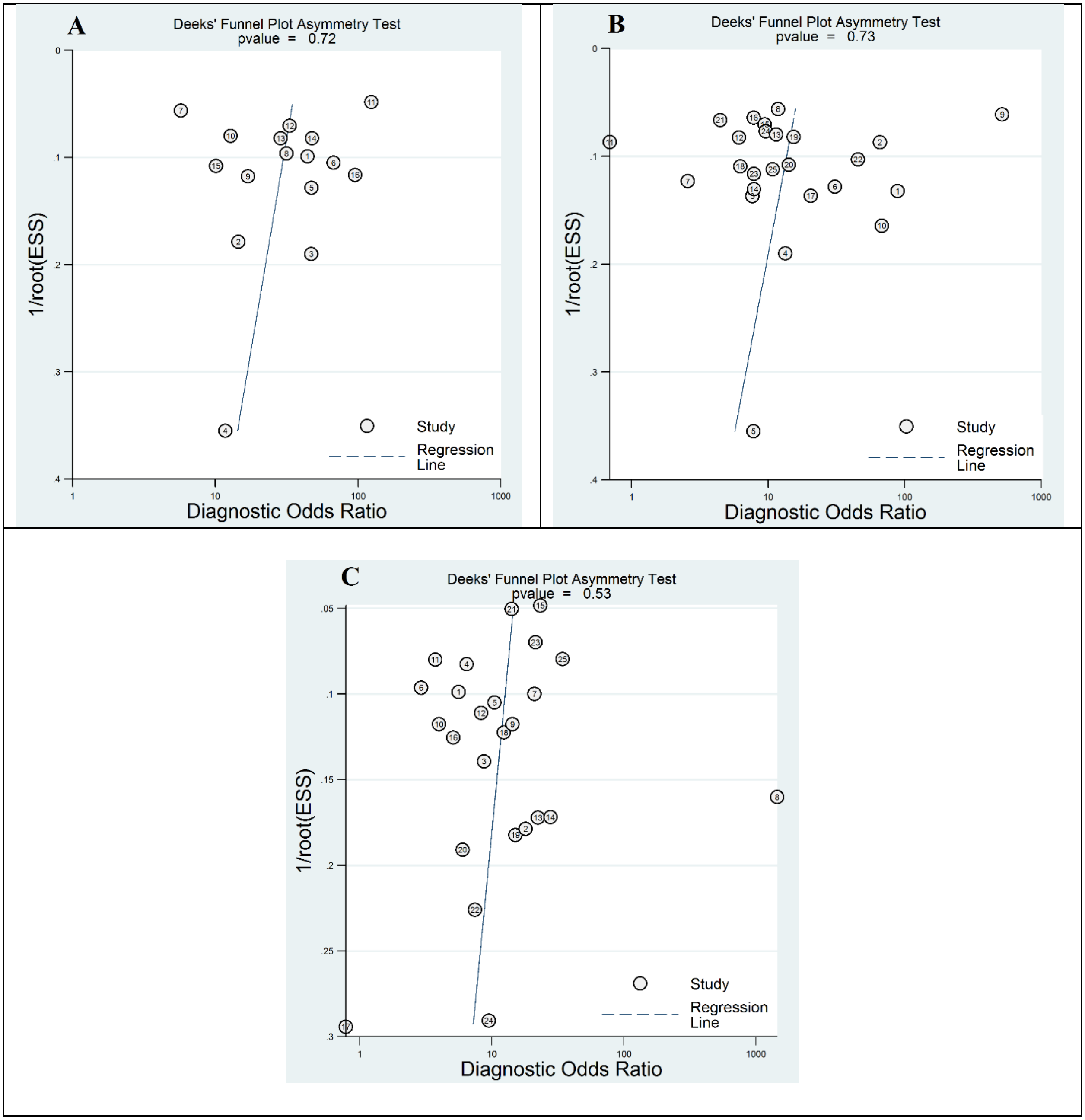


**Supplemental Figure 1.** Deeks' funnel plot asymmetry test for CRC biomarkers. **A:** Immunochemical Fecal occult blood tests; **B:** pyruvate kinase-M2; **C:** fecal calprotectin.

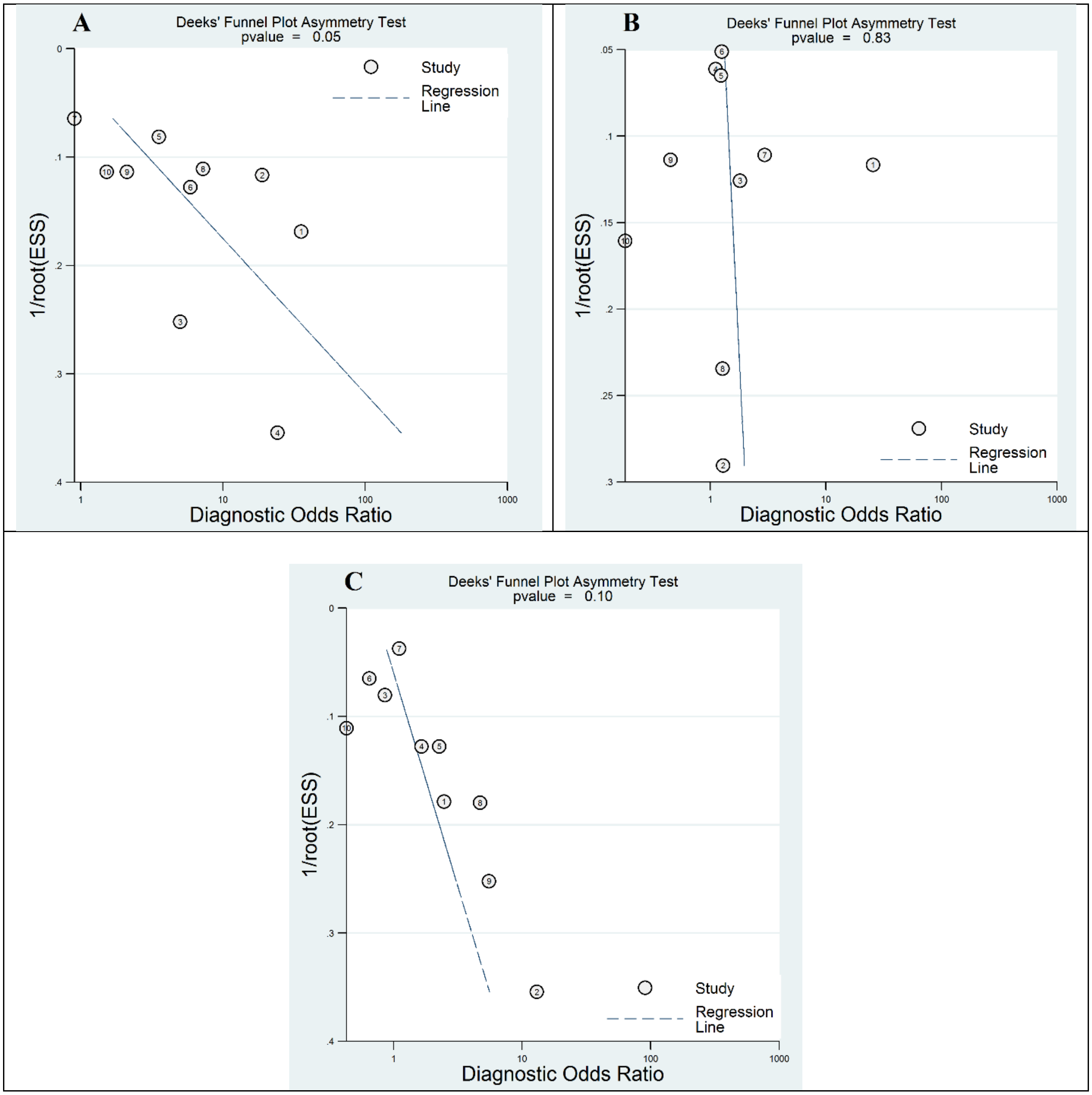


**Supplemental Figure 2.** Deeks' funnel plot asymmetry test for AA biomarkers. **A:** Immunochemical Fecal occult blood tests; **B:** pyruvate kinase-M2; **C:** fecal calprotectin.

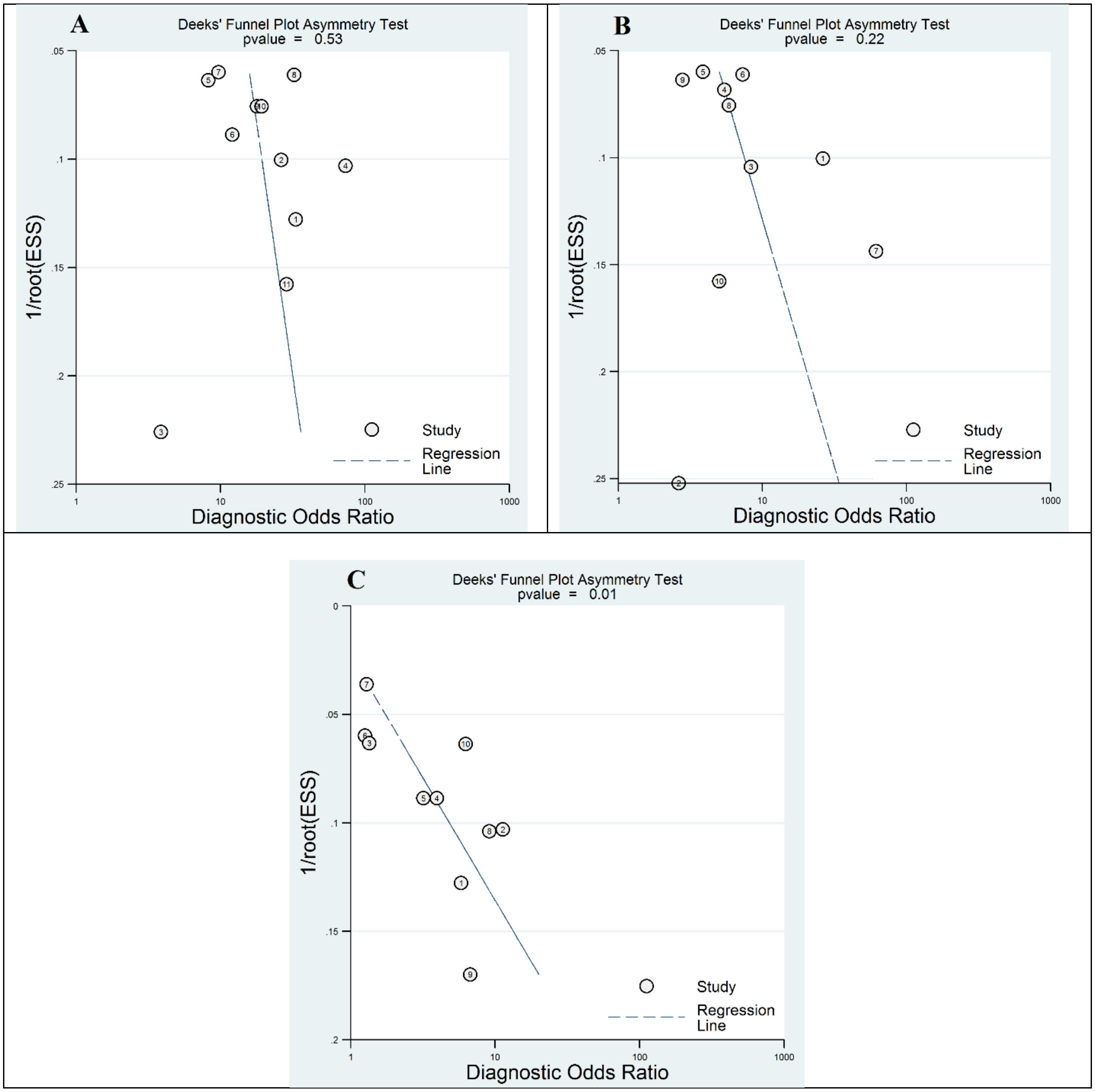


**Supplemental Figure 3.** Deeks' funnel plot asymmetry test for AN biomarkers. **A:** Immunochemical Fecal occult blood tests; **B:** pyruvate kinase-M2; **C:** fecal calprotectin.
